# Unilaterale Implantation einer neuen Intraokularlinse mit erweiterter Tiefenschärfe bei einem jungen Patienten

**DOI:** 10.1007/s00347-020-01285-1

**Published:** 2020-12-10

**Authors:** Isabella D. Baur, Gerd U. Auffarth, Grzegorz Łabuz, Christian S. Mayer, Ramin Khoramnia

**Affiliations:** International Vision Correction Research Centre (IVCRC) und David J Apple International Laboratory for Ocular Pathology, Universitäts-Augenklinik Heidelberg, Im Neuenheimer Feld 400, 69120 Heidelberg, Deutschland

## Falldarstellung

### Anamnese

Ein 46-jähriger Patient stellte sich mit (extern bereits diagnostizierter) einseitiger Katarakt und zunehmender Visusminderung seit wenigen Monaten in unserer Spezialsprechstunde für refraktive Chirurgie vor. Ein Trauma war dem Patienten nicht erinnerlich. Auch Augenoperationen, okuläre Erkrankungen oder Allgemeinerkrankungen sowie Medikamenteneinnahme wurden verneint. In der Familienanamnese war keine Katarakt im frühen Alter bekannt. Der Patient berichtete, bisher keine Brille getragen zu haben, und hatte einen starken Wunsch nach Brillenunabhängigkeit im Alltag. Aufgrund seiner beruflichen Tätigkeit als Angestellter eines Flughafens, die einen hohen Anteil an Nachtarbeit beinhaltet, hatte der Patient sehr hohe Ansprüche an nebenwirkungsarmes Sehen. Der Patient gab an, dass er ggf. auch eine Lesebrille akzeptieren würde, falls dies, evtl. auch im langfristigen Verlauf, erforderlich würde.

### Befund

In der Spaltlampenuntersuchung zeigten sich ein regelrechter Befund mit klarer Linse am rechten Auge und eine hintere Schalentrübung am linken Auge bei ansonsten regelrechtem Befund. Fundoskopisch zeigte sich beidseits ein Normalbefund. Die wichtigsten präoperativen Visusdaten sind in Tab. 1 zusammengefasst.

**Table 1:** 

Auge	Subjektive Refraktion	UDVA (LogMAR)		CDVA (LogMAR)		DCNVA (LogMAR)	
*Rechts*	+0,25/plan/–	−0,14	−0,14	−0,14	−0,14	−0,04	−0,04
*Links*	−0,25/−0,25/105°	+0,44		+0,24		+0,70	

*UDVA* Unkorrigierter Fernvisus, *CDVA* bestkorrigierter Fernvisus, *DCNVA* fernkorrigierter Nahvisus

### Diagnose

Es ergab sich aus der Anamnese und klinischen Untersuchung kein Hinweis auf die Ursache der einseitigen Katarakt. Die Konstellation der einseitigen Erkrankung bei einem jungen Patienten konnte aber am ehesten einer Cataracta traumatica oder Cataracta complicata (z. B. nach nicht mehr erinnerlicher Steroidgabe) zugeordnet werden.

### Therapie und Verlauf

Der Patient wurde ausführlich über die verschiedenen Typen von Intraokularlinsen (IOL), unter anderem über multifokale Intraokularlinsen, Linsen mit erweiterter Tiefenschärfe („extended depth of focus“ [EDoF]) und monofokale IOL informiert. Potenzielle Vorteile und Nebenwirkungen der verschiedenen Optionen wurden mit dem Patienten besprochen. Der Patient wurde über den Verlust der Akkommodation und auch mögliche Komplikationen wie eine intraoperative Kapselruptur oder einen Zonuladefekt bei ggf. traumatisch bedingter Katarakt mit der daraus resultierenden Notwendigkeit, eine monofokale Linse zu implantieren, aufgeklärt. Mit dem Patienten wurde außerdem ausführlich erörtert, dass die Verträglichkeit von IOL mit Sonderfunktion bei nur unilateraler Implantation eingeschränkt sein könnte. Nach sorgfältiger Abwägung entschied sich der Patient für die Implantation einer EDoF-Linse, da diese Option seinen Anforderungen am besten entsprach.

Die optische Biometrie wurde mit dem IOL Master 700 (Carl Zeiss Meditec, Jena, Deutschland) durchgeführt. Die Ergebnisse sind in Tab. 2 zusammengefasst. Die erforderliche Linsenstärke wurde anhand der Haigis-Formel ausgewählt, wobei die Linse mit der geringsten negativen Abweichung von der emmetropen Zielrefraktion ausgewählt wurde.

**Table 2:** 

Auge	Achslänge (mm)	Hornhautradien (mm)		Kornealer Astigmatismus (dpt)	Vorderkammertiefe (mm)
		R1	R2		
*Rechts*	23,95	7,80	7,67	−0,72	3,79
*Links*	23,71	7,57	7,54	−0,20	4,27

Um das refraktive Ergebnis zu optimieren, wurde die Operation unter Einsatz des Femtosekundenlasers (LensX Laser, Alcon, Fort Worth, TX, USA) und eines digitalen Markierungssystems (Verion, Alcon, Fort Worth, TX, USA) durchgeführt. Nach erfolgter Phakoemulsifikation wurde am linken Auge eine AcrySof IQ Vivity IOL (Alcon, Fort Worth, TX, USA) in der Stärke +20,0 dpt implantiert.

Die AcrySof IQ Vivity IOL ist eine einstückige hydrophobe Acrylat-IOL mit einem Gesamtdurchmesser von 13,0 mm und einer optischen Zone von 6,0 mm Durchmesser. Die Ausdehnung des Sehbereichs auf den Zwischenbereich wird durch die nichtdiffraktive Wavefront-Shaping-Technologie (X-Wave-Technologie) erreicht. Die AcrySof IQ Vivity weist im zentralen 2,2-mm-Bereich zwei Übergangselemente auf. Das erste Übergangselement streckt die Wellenfront, wodurch ein kontinuierlicher Fokusbereich entsteht. Allerdings wird das Licht in beide Richtungen gestreckt, also in die myope und in die hyperope Richtung. Das Licht in hyperoper Richtung befindet sich hinter der Netzhaut und wäre so nicht zu nutzen. Daher verschiebt das zweite Übergangselement die Wellenfront nach vorne, wodurch das Licht von der hyperopen Richtung in die myope Richtung verschoben wird, sodass die gesamte Lichtenergie genutzt wird.

**Figure 1:**
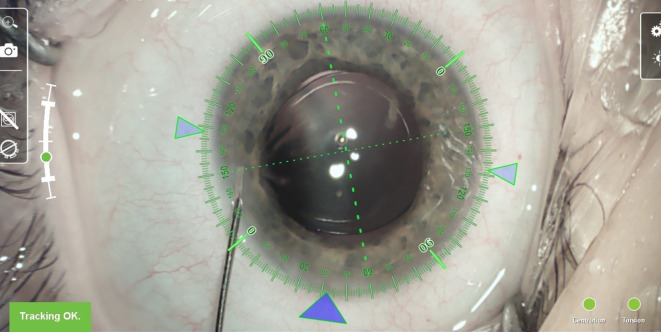

Der Hauptschnitt wurde mithilfe des Markierungssystems zur Korrektur des minimal vorbestehenden kornealen Zylinders bzw. zur Minimierung eines chirurgisch induzierten Astigmatismus auf 42° ausgerichtet. Die Abb. 1 zeigt ein intraoperatives Bild. Der intra- und postoperative Verlauf gestalteten sich komplikationslos.

**Figure 2:**
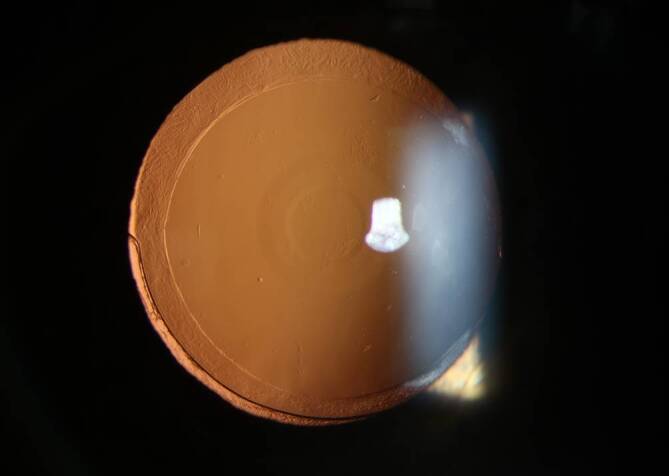

Bei einer Verlaufskontrolle drei Monate postoperativ konnten wir am linken Auge ein sehr gutes funktionelles Ergebnis für die Ferne und den mittleren Nahbereich (80 cm) feststellen. Die wichtigsten postoperativen Visusergebnisse sind in Tab. 3 zusammengefasst. Die Abb. 2 zeigt ein postoperatives Spaltlampenbild der AcrySof IQ Vivity IOL, auf dem die nichtdiffraktive Optik gut zu erkennen ist.

**Table 3:** 

Auge	Subjektive Refraktion	UDVA (LogMAR)		CDVA (LogMAR)		DCIVA (LogMAR)		DCNVA (LogMAR)	
*Rechts*	+0,25/plan/–	−0,12	−0,12	−0,12	−0,14	−0,16	−0,16	−0,04	−0,04
*Links*	Plan/−0,25/120°	−0,08		−0,10		−0,08		+0,20	

*UDVA* Unkorrigierter Fernvisus, *CDVA* bestkorrigierter Fernvisus, *DCIVA* fernkorrigierter Intermediärvisus, *DCNVA* fernkorrigierter Nahvisus

**Figure 3:**
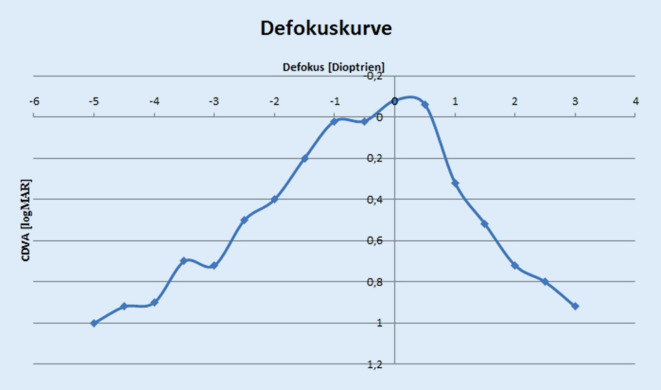

Die Defokuskurve zeigte einen Visus von 0,20 LogMAR oder besser zwischen −1,5 dpt und +0,75 dpt, was insgesamt einem Defokus von +2,25 dpt und einem funktionellen Defokus von etwa 1,5 dpt entspricht. Die Abb. 3 zeigt die monokulare Defokuskurve drei Monate postoperativ.

**Figure 4:**
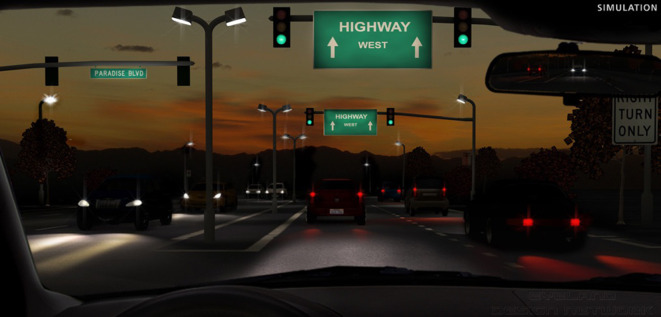

Der Patient wurde gebeten, photische Phänomene mit einem Halo & Glare-Simulator (Eyeland-Design Network GmbH, Vreden, Deutschland) zu bewerten. Der Simulator ermöglicht die Wahl zwischen drei Typen von Halos (klassischer Halo, Starburst und irregulärer Halo). Die Größe und Intensität der Halos kann entsprechend der eigenen Wahrnehmung jeweils auf einer Skala von 0 bis 100 eingestellt werden. Außerdem kann Glare in 2 verschiedenen Formen hinzugefügt werden (klassischer Glare und asymmetrischer Glare) und in der Größe und Intensität variiert werden. Drei Monate postoperativ berichtete der Patient, weder Halos noch Glare wahrzunehmen. Die Abb. 4 zeigt das Ergebnis der Simulation.

## Diskussion

Der Patient erreichte ein sehr gutes funktionelles Ergebnis für die Ferne und den Intermediärbereich und berichtete, keinerlei photische Phänomene wahrzunehmen. Dementsprechend war der Patient sehr zufrieden mit dem postoperativen Ergebnis. Bei der Planung der Operation musste berücksichtigt werden, dass nur an einem Auge eine Indikation zur Kataraktoperation bestand. Zudem legte der Patient großen Wert auf Brillenunabhängigkeit, und insgesamt bestand auch berufsbedingt ein hoher Anspruch an die optische Qualität.

Bei älteren Patienten stellt die senile Katarakt eine häufige Erkrankung dar, deren operative Behandlung mittels Phakoemulsifikation und Hinterkammerlinsenimplantation mittlerweile ein Routineeingriff ist [3, 27]. Bei jungen Erwachsenen hingegen ist eine Katarakt weniger häufig und kann meist einer bestimmten Ursache zugeordnet werden [8]. Zu den möglichen Ursachen gehören u. a. eine langjährige Steroidmedikation [8], Pars-plana-Vitrektomie in der Anamnese [6], kongenitale Katarakt [8], Uveitis [8, 20], Atopie [2] sowie myotone Dystrophie [29] oder ein stumpfes oder perforierendes Bulbustrauma [7]. Je nach Ursache kann auch eine einseitige Katarakt vorliegen. Falls eine Ursache eruiert werden kann, können sich daraus Hinweise auf die Visusprognose und mögliche Komplikationen ergeben. Das Patientenalter spielt auch bei der Wahl einer geeigneten Intraokularlinse eine Rolle, da junge Patienten zumeist nicht an das Tragen einer Lesebrille oder Gleitsichtbrille gewöhnt sind und daher tendenziell einen starken Wunsch nach Brillenunabhängigkeit haben. Zudem stehen junge Erwachsene meist im Berufsleben, und die Anforderungen an das Sehen im Arbeitsalltag müssen bei der operativen Versorgung berücksichtigt werden.

Trifokale Optiken ermöglichen die größte Brillenunabhängigkeit für die Ferne, Nähe und den Intermediärbereich [4, 13, 21]. Sie sind monofokalen Intraokularlinsen im Nah- und Intermediärbereich und bifokalen Linsen im Intermediärbereich überlegen [16, 39]. Die Datenlage zur einseitigen Implantation von multifokalen Intraokularlinsen ist allerdings begrenzt. Jedoch legen die vorhandenen Berichte nahe, dass ein optimales Ergebnis nur nach bilateraler Implantation erreicht werden kann. In einer Studie von Cionni et al. wurde die gleiche diffraktive multifokale Intraokularlinse (AcrySof SN60D3 ReSTOR IOL, Alcon, Fort Worth, TX, USA) entweder unilateral oder bilateral implantiert, und die beiden Patientengruppen wurden miteinander verglichen. Es zeigte sich ein signifikant besseres Ergebnis nach bilateraler Implantation, und auch die Patientenzufriedenheit war in der Gruppe der Patienten, die sich der bilateralen Implantation der multifokalen Linse unterzogen hatten, signifikant höher [12]. Auch beim Binokularsehen konnte ein Unterschied zwischen Patienten nach unilateraler bzw. bilateraler Implantation nachgewiesen werden. In einer Studie von Häring et al. zeigte sich eine signifikant höhere Rate von Aniseikonien sowohl im Fern- als auch im Nahbereich nach unilateraler Implantation einer refraktiven Multifokallinse im Vergleich zu den Patienten nach binokularer Implantation der gleichen Linse [18]. Shoji et al. zeigten, dass die Brillenunabhängigkeit nach beidseitiger Implantation einer refraktiven Multifokallinse höher ist als nach einseitiger Implantation [32]. Unter bestimmten monochromatischen Lichtbedingungen kann die Leistung einer diffraktiven Linse reduziert sein, da die Funktion der diffraktiven Optik von der Wellenlänge des Lichts abhängt [22, 24]. Diffraktive multifokale Intraokularlinsen können das Kontrastsehen negativ beeinflussen und Dysphotopsien wie Lichtringe (Halos) und Blendung (Glare) hervorrufen [10]. Dies ist auf das optische Prinzip der diffraktiven multifokalen Linsen zurückzuführen: Das einfallende Licht wird zwischen verschiedenen Brennpunkten aufgeteilt, und es entstehen sich überlagernde Bilder, wobei nur ein Bild auf der Netzhaut scharf abgebildet wird [33]. Dies erklärt sowohl die reduzierte Kontrastempfindlichkeit als auch die Wahrnehmung von Halos. Die Streuung des Lichts an den diffraktiven Ringen kann zu vermehrter Blendung führen [9]. Cionni et al. beobachteten keinen statistisch signifikanten Unterschied zwischen dem Auftreten von Halos und Blendung bei Patienten nach bilateraler im Vergleich zu unilateraler Implantation einer diffraktiven multifokalen IOL [12].

Unter Berücksichtigung der genannten Faktoren sollte die einseitige Implantation einer trifokalen Intraokularlinse sorgfältig überdacht werden. Vergleicht der Patient die unterschiedlichen Seheindrücke an beiden Augen, könnten die photischen Phänomene als störend empfunden werden. Zudem kann das funktionelle Ergebnis, das nach einseitiger Implantation einer multifokalen IOL erreicht werden kann, nicht zufriedenstellend sein.

Die Hoffnung scheint berechtigt zu sein, dass bei einseitiger Implantation einer nichtdiffraktiven EDoF-IOL eine bessere Verträglichkeit erwartet werden kann. EDoF-IOL erweitern den Sehbereich des Patienten von der Ferne bis zum mittleren Nahbereich. Um eine erweiterte Tiefenschärfe zu erzeugen, können verschiedene optische Prinzipien eingesetzt werden, darunter auch diffraktive und refraktive Linsendesigns sowie Lochblendenoptiken [1, 9, 11, 25, 35–37]. Die AcrySof IQ Vivity IOL erzeugt die erweiterte Schärfentiefe hingegen durch eine Optik, die die Form der Wellenfront verändert. Da es sich bei EDoF-Linsen um eine heterogene Klasse von Intraokularlinsen handelt, ist die Zuordnung zu dieser Kategorie nicht immer eindeutig. Daher hat die American Academy of Ophthalmology Kriterien definiert, um die Klassifizierung einer IOL als „EDoF-IOL“ zu erleichtern und zu standardisieren [28]. Dazu soll eine EDoF-Linse immer mit einer monofokalen Intraokularlinse verglichen werden. Zu den vorgeschlagenen Kriterien gehört, dass der bestkorrigierte Fernvisus vergleichbar mit der monofokalen Kontrolllinse ist und gleichzeitig der fernkorrigierte Intermediärvisus besser als bei der Kontrolllinse ist. Die Defokuskurve soll einen Defokus aufweisen, in dem der Visus 0,2 LogMAR oder besser ist, der mindestens 0,5 dpt größer ist als bei der monofokalen Linse [28]. Eine klinische Studie, die im Rahmen der Zulassung durch die Food and Drug Administration (FDA) durchgeführt wurde, bestätigte diese Eigenschaften für die AcrySof IQ Vivity IOL [41].

Liu et al. verglichen trifokale (PanOptix [Alcon, Fort Worth, TX, USA], FineVision [PhysIOL, Liège, Belgien] und Lisa tri 839MP [Zeiss, Oberkochen, Deutschland]) und monofokale IOL (Tecnis ZCB00 [Johnson & Johnson, New Brunswick, New Jersey, USA] und AcrySof SN60WF [Alcon, Fort Worth, TX, USA]) im Rahmen einer Metaanalyse mit einer diffraktiven EDoF-Intraokularlinse, der Tecnis Symfony ZXR00 (Johnson & Johnson, New Brunswick, New Jersey, USA) [26]. Für die EDoF-Linse zeigten sich im Intermediär- und Nahbereich bessere Ergebnisse als für die monofokalen IOL, im Nahbereich war die untersuchte EDoF-Linse den trifokalen Linsen jedoch unterlegen. Die EDoF IOL (Tecnis Symfony ZXR00) zeigte im Vergleich zu den monofokalen Linsen eine reduzierte Kontrastempfindlichkeit [26]. Im Vergleich zu den trifokalen Intraokularlinsen war die EDoF-Linse aber bezüglich der Kontrastsensitivität überlegen. Diese Studie zeigte keinen Unterschied zwischen der EDoF-IOL (Tecnis Symfony ZXR00) und den trifokalen Linsen bezüglich der Brillenunabhängigkeit [26]. Arbeiten zeigten aber, dass die spektrale Abhängigkeit dieser diffraktiven EDoF IOL die Sehschärfe und die Kontrastempfindlichkeit beeinflussen kann [22, 23, 38]. Da EDoF-IOL unterschiedliche optische Prinzipien aufweisen, unterscheidet sich das Ausmaß der photischen Phänomene teilweise deutlich. Bei bestimmten EDoF-IOL (Mini Well; SIFI, Catania, Italien) wurden weniger Dysphotopsien im Vergleich zu trifokalen IOL beobachtet [5, 17, 31]. Diffraktive EDoF-Linsen (z. B. Tecnis Symfony ZXR00) scheinen hingegen mit einem vergleichbaren Maß an photischen Phänomenen wie trifokale Intraokularlinsen assoziiert zu sein [14, 15, 26, 30]. Bei der AcrySof IQ Vivity IOL, die auf der nichtdiffraktiven Wavefront-Shaping-Technologie basiert, zeigen die Daten der Studie zur FDA-Zulassung hingegen nur geringfügig ausgeprägte photische Phänomene [41].

Bei unserem Patienten konnte die Diagnose einer traumatischen Katarakt nicht ausgeschlossen werden. Daher musste die Möglichkeit berücksichtigt werden, dass es aufgrund eines klinisch noch nicht manifesten Zonuladefekts im Verlauf zu einer Dezentrierung der implantierten Intraokularlinse kommen könnte. Die optische Qualität von bifokalen und trifokalen IOL hängt jedoch stark von einer perfekten Zentrierung der IOL ab [19, 34, 40]. Die Toleranz gegenüber Dezentrierung ist bei EDoF-IOL höher als bei multifokalen Linsen [42], was ebenfalls für die Implantation einer EDoF-Linse sprach.

## Fazit für die Praxis

Wir beobachteten sehr gute funktionelle Ergebnisse für die Ferne und den mittleren Nahbereich nach einseitiger Implantation der AcrySof IQ Vivity IOL bei einem jungen Patienten mit sehr hohen Anforderungen an eine gute Sehqualität trotz des Wunsches nach Brillenunabhängigkeit. Der Patient berichtete, keinerlei photische Phänomene wahrzunehmen. Auch die nur unilaterale Implantation wurde gut toleriert.
